# 

**Published:** 1887-01

**Authors:** 


					﻿Kvta» i ittSar Putt	• -i ¿uw, Xoa-m.- y* ¿¿»«Ia&u-' .Va-! jfirfter
Vol. II.
JANUARY, xSS;
No. 7
DANIEL'S
MEDICAL
JOURNAL
F E. DANIEL.. M D . Editor and Publisher, AUSTIN. TEXAS.
Aortliern ontf*. SSi? ®*t!bert street, PhilattcJphia.
« Boecki!a.yn, Steam Book and Job Printer,	’iicxTs.
				

